# Unravelling the Genetic Basis of Primary Aldosteronism

**DOI:** 10.3390/nu13030875

**Published:** 2021-03-08

**Authors:** Niki Mourtzi, Amalia Sertedaki, Athina Markou, George P. Piaditis, Evangelia Charmandari

**Affiliations:** 1 Division of Endocrinology, Metabolism and Diabetes, First Department of Pediatrics, National and Kapodistrian University of Athens Medical School, “Aghia Sophia” Children’s Hospital, 11527 Athens, Greece; nikimourtzi23@gmail.com (N.M.); aserted@med.uoa.gr (A.S.); 2 Department of Endocrinology and Diabetes Center, G. Gennimatas General Hospital, 11527 Athens, Greece; amarkouuk@yahoo.co.uk (A.M.); edk-pgna@otenet.gr (G.P.P.); 3 Division of Endocrinology and Metabolism, Center of Clinical, Experimental Surgery and Translational Research, Biomedical Research Foundation of the Academy of Athens, 11527 Athens, Greece

**Keywords:** primary aldosteronism, hypertension, cardiovascular disease, genetic causes of primary aldosteronism

## Abstract

Primary aldosteronism (PA), a condition characterized by autonomous aldosterone hypersecretion, constitutes the most common cause of secondary hypertension. Over the last decade, major breakthroughs have been made in the field of genetics underpinning PA. The advent and wide application of Next Generation Sequencing (NGS) technology led to the identification of several somatic and germline mutations associated with sporadic and familial forms of PA. Somatic mutations in ion-channel genes that participate in aldosterone biosynthesis, including *KCNJ5*, *CACNA1D*, *ATP1A1,* and *ATP2B3*, have been implicated in the development of aldosterone-producing adenomas (APAs). On the other hand, germline variants in *CLCN2*, *KCNJ5*, *CACNA1H*, and *CACNA1D* genes have been implicated in the pathogenesis of the familial forms of PA, FH-II, FH-III, and F-IV, as well as PA associated with seizures and neurological abnormalities. However, recent studies have shown that the prevalence of PA is higher than previously thought, indicating the need for an improvement of our diagnostic tools. Further research is required to recognize mild forms of PA and to investigate the underlying molecular mechanisms.

## 1. Introduction

Hypertension is a major risk factor for the development of cardiovascular disease and its associated morbidity and mortality, and has been identified as the third leading cause of disability-adjusted life-years [1]. The global prevalence of hypertension for the year 2000 has been estimated to be 26.4% of the world population (976 million) and is expected to increase to 29% (1.56 billion) by 2025, with tremendous implications for the health care system [2].

Primary Aldosteronism (PA), a condition characterized by autonomous aldosterone overproduction, was first described by Conn in 1955 and was considered to be a rare cause of hypertension with a prevalence of less than 1% among hypertensive patients. However, following the introduction of plasma aldosterone concentration to plasma renin activity ratio (ARR) as a screening test in clinical practice, it became evident that the prevalence of PA is higher than previously thought [3,4,5]. Currently, PA is recognized as the most common cause of secondary hypertension, accounting for 6.1% of hypertensive patients and up to 20% of patients with severe or treatment-resistant hypertension [6,7]. Early identification and treatment of patients with PA is of paramount importance because they have a higher risk of stroke, myocardial infarction, and atrial fibrillation than patients with essential hypertension matched for age, gender, and blood pressure [8].

Over the last years, with the advent of Next Generation Sequencing (NGS), major advances have been made in our understanding of the molecular mechanisms underlying autonomous aldosterone overproduction in both sporadic and familial forms of PA. The purpose of this review is to provide an update on the current knowledge on the genetics of PA.

## 2. Evidence Favoring a Higher Prevalence of PA

The traditional approach in the diagnosis of PA involves an initial screening procedure to identify patients with higher probability for PA (Table 1) [9], followed by the application of aldosterone-to-renin ratio (ARR) test to detect elevated concentrations of aldosterone. Patients with positive ARR undergo a confirmatory test (e.g., oral-sodium loading, saline-infusion test, fludrocortisone suppression test, captopril test) in order to confirm or exclude autonomous aldosterone secretion independent of the renin-angiotensin system (RAAS), and subsequently an adrenal computed tomography (CT) scan to investigate the existence of an adrenal mass (bilateral or unilateral) [10]. Following this approach, Monticone et al. reported a prevalence of PA between 3.9% and 11.8%, depending on the severity of hypertension [11].

However, compelling evidence suggests that PA is much more prevalent than previously thought, with a large number of PA cases remaining undiagnosed [12,13]. This is mainly attributed to the fact that current diagnostic tests based on plasma aldosterone and renin concentrations may be insufficient to identify mild forms of PA, especially if based on the arbitrary conventional ARR thresholds [11]. Piaditis and others [13,14] have shown that when PA screening is extended to all hypertensive patients, irrespectively of the ARR, the PA prevalence is 3–5 times higher than previously determined, ranging between 16% and 31% across the spectrum of mild to severe hypertension [13,14]. Critically, PA has been documented in 11% and 13% of apparently healthy normotensive and normokalemic individuals that would escape diagnosis when the current guidelines are adopted or followed [13,14]. This becomes extremely prominent when considering the fact that 85% of a normotensive cohort with subclinical PA developed hypertension at 5-years of follow-up [15]. In line with these studies, administration of mineralocorticoid receptor antagonists (MRAs) has been shown to successfully control the blood pressure of patients with apparent essential hypertension, with their efficacy to be positively correlated with the levels of within the “normal” range aldosterone excess [16].

In light of these findings, Markou et al. investigated the role of ACTH, which has been predominantly regarded only as a minor regulator of aldosterone secretion in the development of PA. More specifically, they modified the standard fludrocortisone-saline suppression test (FST) by adding 2 mg of dexamethasone on the last day of testing (fludrocortisone suppression test, FDST) in order to eliminate the ACTH effect on aldosterone secretion [17]. Strikingly, after applying the FDST test to a cohort of hypertensive patients with normal ARR result and adrenal morphology, 31% of this cohort was diagnosed with PA and successfully treated [14]. In a subsequent study, we identified two novel germline mutations in the *KCNJ5* gene in a cohort of patients without PA and normal adrenal CT, who exhibited ACTH-dependent aldosterone hypersecretion and responded to treatment with MRAs [18,19]. This work further expanded the clinical spectrum associated with *KCNJ5* mutations and highlighted the importance of determining the genetic etiology of aldosterone hypersecretion in mild forms of primary aldosteronism towards earlier and targeted treatment.

## 3. Genetics of Primary Aldosteronism

PA can be either sporadic or familial. Sporadic cases of PA, Bilateral Adrenal Hyperplasia (BAH), and Aldosterone-Producing Adenomas (APAs), constitute the majority of PA cases (95%).

On the other hand, only 5% of cases are familial, and four familial forms of PA, FH-I, FH-II, FH-III, and FH-IV, which are inherited in an autosomal dominant manner, have been recognized to date [20].

## 4. Genetics of Sporadic Forms of Primary Aldosteronism 

Prior to the advent of NGS, studies on sporadic forms of PA primarily focused on genetic variants influencing the susceptibility to PA or acting as phenotype modifiers, such as *CYP11B2*, a-Adducin, and Bradykinin B2 Receptor gene polymorphism [21]. The development and widespread use of NGS shifted our approach and led to the identification of several somatic mutations in APAs that not only identified APA-driver genes but also furthered our understanding of the molecular mechanisms of aldosterone production regulation [21]. The majority of the somatic mutations in sporadic forms of PA reported to date (Table 2) have been identified into well-characterized ion channels and ATPases genes involved in aldosterone biosynthesis pathway, including potassium channels (*KCNJ5*), calcium channels (*CACNA1D*, *CACNA1H*), and ATPases (*ATP1A1*, *ATP2B3*) [22]. More recently, somatic mutations in *CTNNB1*, *PRKACA*, and *ARMC5* genes (Table 2) have been implicated in the pathogenesis of APAs [22].

### 4.1. KCNJ5 Mutations

In 2011, Choi et al. identified two recurrent heterozygous mutations in the *KCNJ5* gene (p.Gly151Arg and p.Leu168Arg) in 8/22 APA samples [23]. Subsequent studies revealed that *KCNJ5* somatic mutations constitute the most frequent genetic defect in APAs, with their prevalence reaching 40–60% [24,25] in Caucasians and 70% in Japanese patients [26]. Furthermore, higher frequencies have been reported in female compared with male patients (67% vs. 44% respectively) [27]. The *KCNJ5* (potassium inwardly rectifying channel, subfamily J, member 5) gene encodes the G protein-activated inward rectifier potassium channels (Kir) 3.4, highly expressed in adrenal gromerulosa cells and important for maintaining the normal resting negative membrane potential of gromerulosa cells by mediating efflux of potassium ions. The mutations identified in APAs are located near the selectivity filter of the channel and abolish the K^+^ selectivity of the channel that results in increased Na^+^ influx, membrane depolarization, and opening of the voltage-gated calcium channels [23]. The rise in intracellular Ca^2+^ concentration triggers the transcription of aldosterone synthase gene, *CYP11B2*, and leads to autonomous aldosterone hypersecretion [23].

Regarding the histological phenotype of *KCNJ5*-positive tumors, they are characterized by zona fasciculata (ZF)–like cell composition and by lower pre-contrast Hounsfield Units (H.U) units than tumors mutated in other genes [28]. *KCNJ5-*positive tumors tend to be larger compared to cases with other somatic mutations or non-mutated cases, often requiring unilateral adrenalectomy [28], and secrete high amounts of the steroid 18-oxocortisol that could serve as a biomarker to determine which APA patients might need surgery [29]. Considering the specific histopathologic phenotypes of *KCNJ5-*positive tumors, it has been suggested that young females with larger tumors and lower H.U units on CT scan, a profile that indicates the presence of *KCNJ5* mutations, may avoid the procedure of adrenal vein sampling prior to surgery [28].

Transcriptomic analysis of APAs revealed that *KCNJ5*-positive tumors tend to exhibit lower levels of *CYP11B2* expression and higher levels of *CYP11B1* than *CACNA1D/ATP1A1/ATP2B3*-positive tumors [30]. The relatively low levels of *CYP11B2* expression in *KCNJ5* tumors is compensated by their large tumor size, explaining the similar plasma aldosterone concentrations in APAs patients with other genetic mutations [31]. In some studies, patients with *KCNJ5-*positive tumors have been associated with a better surgical outcome and postoperative resolution of hypertension with the administration of limited number hypertensive drugs compared to patients with wild type *KCNJ5* channels [32,33,34]. In vitro studies have shown that *KCNJ5-*mutated channels can be inhibited by macrolide antibiotics. Ongoing clinical trials evaluate the clinical effectiveness of two macrolides, clarithromycin, and roxithromycin, in the treatment of PA, and assess whether plasma aldosterone concentrations and blood pressure changes in response to these agents can be predictive of *KCNJ5*-positive tumors [35].

### 4.2. CACNA1D Mutations

By performing exome sequencing, two independent studies reported *CACNA1D* gene somatic mutations in 8% and 5% of APAs studied [36,37]. *CACNA1D* gene encodes for the pore-forming Cav1.3 subunit of the L-type voltage-gated calcium channel that contains 4 repeated domains (I–IV), each with 6 transmembrane segments (S1–S6) and a membrane-associated loop between S5 and S6. The S5 and S6 segments, together with the membrane-associated loop form the pore of the channel [29]. Electrophysiological studies showed that the identified somatic mutations cause an increase in calcium permeability of glomerulosa cells and a concomitant rise in aldosterone production either by shifting the activation to more negative potentials or by reducing the inactivation of the channel [28,29]. Compared to *KCNJ5* mutations, *CACNA1D* mutations are more scattered throughout the protein and are detected in different regions involved in activation (S6 segment) and voltage sensing (S4 and S5 segment); therefore, a thorough sequencing of *CACNA1D* gene is recommended in patients with APAs. In some but not all studies, *CACNA1D* mutations have been linked with a glomerulosa-like phenotype [36,38], suggesting that these tumors derive from zona glumerolosa (ZG) cells [37]. In comparison with *KCNJ5*-positive tumors, *CACNA1D-*positive tumors are significantly smaller in size with higher H.U units on CT scan [28,37], and they have negative expression of *CYP11B1* and higher expression of *CYP11B2* [39]. In the study by Kitamoto et al. [40], it was reported that *CACNA1D* APAs with a predominant zona fasciculata composition display moderate autonomous aldosterone production compared to tumors with ATPase mutations [40]. Patients with *CACNA1D* mutations are more often males [36], older, and with a milder phenotype of hypokalemia than patients with tumors of different genetic background [33]. Calcium channel blocker administration has been proposed as a potential targeted treatment of this distinct cohort of patients, apart from the surgical resection of the affected adrenal glands, in severe cases [37].

### 4.3. ATPA1 and ATPA2B3 Mutations

Beuschein et al. [41] identified mutations in two members of the P-type ATPase gene family, namely *ATP1A1* (coding the a1 subunit of the Na(+)/K(+) ATPase) and *ATP2B3* (coding for the plasma membrane Ca^2+^-ATPase, type 3) in three and two of the nine APAs studied, respectively [30]. Subsequently, the investigators demonstrated that variants clustered in the protein ion-binding domains of *ATP1A1* and *ATP2B3* cause increased sodium and protons conductance, leading to cell depolarization and enhanced aldosterone production, a mechanism similar to *KCNJ5* mutations [28,42]. APAs with ATPase mutations, despite their relatively small sample size compared to *CACNA1D* adrenal lesions, have been associated with a more severe PA phenotype, marked by increased aldosterone production [40]. Furthermore, the number of hypertensive medications required for controlling the BP of patients with ATPase mutations has been reported to be higher than that of patients with *KCNJ5-*positive mutations [43]. Similar to *CACNA1D*-positive tumors, it has been shown by several studies that *ATP1A1/ATP2B3*-positive tumors display a ZG-like histological appearance [41,44], and they are characterized by strong and weak expression of *CYP11B2* and *CYP11B1*, respectively [41,44].

### 4.4. CTNNB1, PRKACA, ARMC5, and CLCN2 Mutations

Activating mutations in *CTNNB1*, the gene encoding beta-catenin, the final effector of WNT pathway, were reported in APAs by Åkerström et al. [45]. Although, the exact mechanism by which mutations in beta-catenin can lead to PA is unknown, this finding supports that sustained activation of WNT pathway leads to the formation of aldosterone-producing lesions [32]. *CTNNB1*-positive tumors have larger adrenal lesion diameters compared to non-mutated tumors and are more prevalent in females, similar to *KCNJ5*-positive tumors [28,45]. Rare causes of APAs (1.6%) are somatic mutations in *PRKACA* (catalytic subunit of protein kinase A) gene, a gene mainly implicated in cortisol-producing adenomas. However, the exact pathophysiologic process leading to APA formation has not been fully elucidated for a group of cortisol co-secreting APAs [46]. The identified patients bearing *PRKACA* somatic mutations were treated with unilateral adrenalectomy, whereas postoperative administration of a limited number of antihypertensive drugs successfully controlled their BP. Germline variants predicted as damaging in Armadillo Repeat Containing Protein 5 (*ARMC5*) gene have been reported in African-American patients [47] with sporadic PA but not in Caucasian patients with PA [48]. Patients with tumors carrying *ARMC5* variants presented with varying levels of hyperaldosteronism, low-renin hypertension, and decreased expression of *CYP11B2* [47]. The lower expression of CYP11B2 was possibly compensated by the increased adrenocortical mass, leading to hyperaldosteronism [47]. Recently, a mutation in *CLCN2* gene (p.Gly24Asp), associated with FH-II, was identified in a patient with APA [49].

## 5. Genetics of Familial Forms of Primary Aldosteronism 

**Figure 1:**
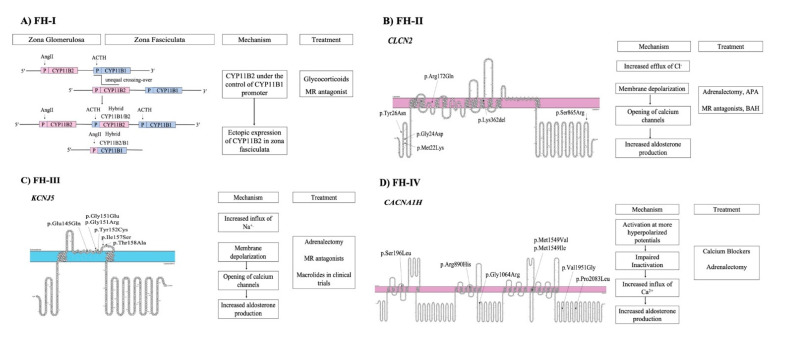
Pathogenetic mechanisms involved in familial forms of Primary Aldosteronism and treatment (**A**) FH-I is caused by an unequal crossover between *CYP11B1* and *CYP11B2* genes. (**B**) FH-II is caused by mutations in *CLCN2* gene that lead to opening of calcium channels. (**C**) FH-III is caused by mutations in *KCNJ5* gene. (**D**) FH-IV is caused by mutations in *CACNA1H* gene.

Familial hyperaldosteronism is a rare cause of PA and is categorized into four types, each one characterized by specific clinical features and genetic causes (Figure 1) (Table 3). However, a considerable proportion of cases with early-onset PA remains unsolved, indicating that new genes responsible for FH remain to be identified [50].

## 6. Familial Hyperaldosteronism Type I, FH-I

The first familial form of PA, described by Sutherland et al. [51] in 1966, was initially referred to as glucocorticoid-remediable aldosteronism (GRA) because it was treated with dexamethasone. However, it was renamed FH-I after the identification of additional forms of FH. The molecular basis of FH-I was delineated more than two decades after its first description, by the work of Lifton et al. [52], who recognized as its cause the presence of a chimeric gene produced by an unequal crossing-over event between the promoter region of 11-beta hydroxylase gene (*CYP11B1*) that participates in cortisol biosynthesis and the coding regions of aldosterone synthase (*CYP11B2*) gene. As a result, aldosterone is ectopically synthesized in the adrenal zona fasciculata under the regulation of ACTH and not under the renin-angiotensin system (RAS) [39]. The diagnosis of FH-I is based on genetic testing for the presence of the chimeric gene, while the treatment involves MRAs or glucocorticoid administration [7].

### 6.1. Familial Hyperaldosteronism Type II, FH-II

FH-II, first described by Gordon et al. [53] in 1991, constitutes the most common subtype of FH, with a prevalence of 1.2–6% [54], which is characterized by bilateral adrenal hyperplasia and/or the presence of adrenal lesions similar to sporadic PA. A linkage analysis showed an association of FH-II with the chromosome region 7p22 [55]. However, its genetic cause was unknown until recently. In 2018, two independent groups [37,56] reported gain of function mutations in *CLCN2* gene that encodes a voltage-gated chloride channel in kindreds with FH-II. *CLCN2* is highly expressed in zona glomerulosa cells and is activated under hyperpolarized membrane potentials promoting aldosterone biosynthesis. *CLCN2* mutations in FH-II patients cause increased chloride efflux leading to membrane depolarization at resting potential, opening of voltage-gated calcium channels, and enhanced aldosterone production [37,43].

### 6.2. Familial Hyperaldosteronism Type III, FH-III

FH-III was initially described in 2008 in a father and two daughters who presented with early-onset severe hypertension, profound hypokalemia, overproduction of 18-oxocortisol and 18-hydroxycortisol and marked adrenal hyperplasia [57]. Soon thereafter, we reported a new family with FH-III, in which two affected members, a mother, and a daughter, presented with severe PA and massive bilateral adrenal hyperplasia caused by a novel point mutation of the *KCNJ5* gene [58]. Both the index case and her mother presented in childhood with markedly elevated aldosterone concentrations, suppressed plasma renin activity, and early-onset severe hypertension refractory to treatment, which required bilateral adrenalectomy. Sequencing of the *KCNJ5* gene revealed a single, heterozygous guanine to thymine (G to T) substitution at nucleotide position 470, resulting in isoleucine to serine (I to S) substitution at amino acid position 157. This mutation results in loss of ion selectivity, cell membrane depolarization, increased Ca^2+^ entry in adrenal glomerulosa cells, and increased aldosterone synthesis [45]. The genetic cause of FH-III is attributed to germline mutations in *KCNJ5* gene that alter the K^+^ selectivity of the channel [20]. Patients with FH-III exhibit a broad spectrum of phenotypic variability from mild or moderate hypertension responsive to treatment, to resistant hypertension due to massive adrenal hyperplasia [59]. Notably, patients with the same inherited *KCNJ5* mutation (G151R) but with different phenotypes and severity of hyperaldosteronism have been reported, indicating that *KCNJ5* genotype is insufficient to explain phenotypic variability and that other genetic and/or environmental factors may influence the phenotype [46].

### 6.3. Familial Hyperaldosteronism Type IV, FH-IV

In an effort to delineate the genetic cause of PA cases with unknown etiology, Scholl et al. performed exome sequencing of 40 unrelated patients with early-onset hypertension (<10 years) [60]. Five subjects were identified as carriers of a *de novo* germline mutation (p.Met1549Val) in *CACNA1H* gene, encoding the voltage-gated T-type calcium channel Cav3.2. Functional analysis showed that the mutation causes impaired inactivation and activation of the channel at more hyperpolarized potentials, inducing increased Ca^2+^ influx and aldosterone hypersecretion [47]. Subsequently, Danill et al. [48] reported germline *CACNA1H* mutations in PA patients with different forms of PA, from familial hyperaldosteronism to sporadic PA (APA presence) and early-onset hypertension associated with multiple developmental disorders [61]. These findings suggest that *CACNA1H* is a susceptibility gene for PA development with a wide range of phenotypic presentation [48].

### 6.4. PA with Seizures and Neurological Abnormalities (PASNA)

PASNA, a new form of PA characterized by early-onset PA, neurological abnormalities, and seizures, is caused by gain-of-function mutations in *CACNA1D* gene [29]. Two children with PASNA syndrome were found to harbor germline mutations in *CACNA1D* gene, and functional studies demonstrated that these mutations lead to activation of the channel at less depolarized potentials or affect deactivation of the channel [29]. Furthermore, another infant with autism and epilepsy, features similar to the phenotype of PASNA syndrome, was reported to carry a mutation in *CACNA1D* gene [62]. Interestingly, a patient with hyperinsulinemic hypoglycemia but with normal aldosterone concentrations has been reported to be heterozygous for p.G403D change, a mutation previously reported in a PASNA case with PA [63]. It is likely that the presence of genetic and environmental modifiers may account for the phenotypic discordance in the two patients [50].

## 7. Conclusions

During the last decade, the development and wide application of NGS technology led to the identification of several somatic and germline mutations associated with sporadic and familial forms of PA. Notably, all causative genes reported to date are involved in well-characterized pathways regulating aldosterone production, such as potassium and calcium-sensing pathways. These findings do not only define new disease-causing genes and improve our understanding of the pathophysiology behind aldosterone biosynthesis, but also open the door to the development of novel pharmacologic agents for the treatment of patients with PA. However, the clinical approach for the diagnosis of mild forms of PA needs to be updated to allow the early identification of patients with subclinical PA. Finally, it is imperative to unravel the genetic background of PA across its clinical spectrum, from mild to severe, which, in turn, will facilitate the appropriate management and treatment of primary aldosteronism, a much more prevalent syndrome than previously thought.

## Figures and Tables

**Table 1:** The current recommendations of the Endocrine Society for Primary Aldosteronism screening.

The Current Recommendations of the Endocrine Society for Primary Aldosteronism Screening
Sustained blood pressure (BP) above 150/100 mmHg on each of three measurements obtained on different days Hypertension (BP > 140/90 mmHg) resistant, not controlled by three conventional antihypertensive drugs (including a diuretic) Controlled BP (<140/90 mmHg) on four or more antihypertensive drugs Hypertension and spontaneous or diuretic-induced hypokalemia Hypertension and adrenal incidentaloma Hypertension and sleep apnea Hypertension and a family history of early-onset hypertension or cerebrovascular accident at a young age (<40 years). All hypertensive first-degree relatives of a patient with Primary Aldosteronism

**Table 2:** Mutations reported in sporadic primary aldosteronism.

Gene	Location (Hg19 *)	Somatic Mutations	Sporadic Germline Mutations
*KCNJ5*	11:128,761,251-128,790,930	p.R115W	p.R52H
		p.T126R	p.E145Q
		p.A139-F142dup	p.G151R
		p.F140L	p.G151E
		p.144-G145insAI	p.Y152C
		p.E145Q	p.I157S
		p.E145K	p.T158A
		p.E145_E147delinsK	p.G246K
		p.E147Q_T149_I150insTTT	p.E247R
		p.T148I	
		p.T149insR	
		p.T149S	
		p.T149delinsTT	
		p.T149delinsMA	
		p.I150_G151insM	
		p.G151R	
		p.G153-G164dup	
		p.F154C	
		p.I157del	
		p.I57_E159del	
		p.I157K	
		p.T158A	
		p.L168R	
		p.G184E	
		p.G246K	
*CACNA1D*	3:53,529,076-53,846,492	p.V259A	p.I770M
		p.V309A	p.G403R
		p.V401L	p.F747L
		p.G403R	
		p.R619P	
		p.S652L	
		p.L655P	
		p.Y741C	
		p.F747L	
		p.F747C	
		p.F747V	
		p.V979N	
		p.P1336R	
		p.I750M	
		p.R990G	
		p.R993T	
		p.A998I	
		p.A998V	
		p.C1007R	
		p.I1015S	
		p.V1151F	
		p.I1152N	
		p.V1338M	
		p.V1353M	
*ATP1A1*	1:116,915,795-116,947,396	p.G99R	
		p.F100L	
		p.P100_L104del)	
		p.L104R	
		p.V332G	
		p.I955_E960delinsK	
		p.960-963delp.A963S	
*ATP2B3*	X:152,801,580-152,848,387	p.L425_V426del	
		p.V426_V427del	
		p.V424_L425del	
*CTNNB1*	3:41,240,942-41,281,939	p.S33C	
		p.G34R	
		p.A39Efs*3	
		p.S45P	
		p.S45F	
		p.V426G_V427Q_A428_L433del	
*CLCN2*	3:184,063,973-184,079,439	p.Gly24Asp	
*PRKACA*	19:14,202,507-14,228,559	p.His88Asp	
		p.Arg201Cys	
		p.Leu206Arg	
*ARMC5*	16:31,470,317-31,478,488		p.F14Y
			p.L156F
			p.I170V
			p.G323A
			p.R502H
			p.P507L
			p.T643M
			p.P826H
			p.R898W

* Hg19: Human Reference Genome Hg19.

**Table 3:** Clinical characteristics of FH type I, II, III, and IV.

Familial Form of Primary Aldosteronism	Clinical Characteristics
Familial hyperaldosteronism type I, FH-I or glucocorticoid-remediable aldosteronism	Glucocorticoid-sensitive PATreated with dexamethasone administrationAdrenal hyperplasiaLow renin levelsIntracranial aneurysm and hemorrhagic strokeHypokalemiaNormotensive to severely hypertensiveHybrid steroid levels
Familial hyperaldosteronism type II	Phenotype resembling sporadic forms of PAHypokalemiaLow renin levelsGlucocorticoid-insensitive PAAPAs or Adrenal hyperplasia or bothNormotensive to severely hypertensive
Familial hyperaldosteronism type III	Low renin levelsBilateral massive adrenal hyperplasiaGlucocorticoid-insensitive PAHypokalemiaSeverely hypertensive
Familial hyperaldosteronism type IV	Low renin levelsNormal adrenal morphologyWide range of clinical phenotypes

## Data Availability

Data sharing not applicable.
